# The ecosystem-service chain and the biological diversity crisis

**DOI:** 10.1098/rstb.2009.0223

**Published:** 2010-01-12

**Authors:** Harold A. Mooney

**Affiliations:** Department of Biology, Stanford University, Stanford, CA 94305, USA

**Keywords:** ecosystem services, biological diversity crisis, Convention on Biological Diversity

## Abstract

The losses that are being incurred of the Earth's biological diversity, at all levels, are now staggering. The trend lines for future loss are steeply upward as new adverse drivers of change come into play. The political processes for matching this crisis are now inadequate and the science needs to address this issue are huge and slow to fulfil, even though strong advances have been made. A more integrated approach to evaluating biodiversity in terms that are meaningful to the larger community is needed that can provide understandable metrics of the consequences to society of the losses that are occurring. Greater attention is also needed in forecasting likely diversity-loss scenarios in the near term and strategies for alleviating detrimental consequences. At the international level, the Convention on Biological Diversity must be revisited to make it more powerful to meet the needs that originally motivated its creation. Similarly, at local and regional levels, an ecosystem-service approach to conservation can bring new understanding to the value, and hence the need for protection, of the existing natural capital.

## 1. Introduction

The biological diversity of Earth is vast and a source of wonder and inspiration to humans, who are a dominant component of this complex fabric and utterly dependent upon it. Humans, through the ages, have sought to understand the origins, nature and status of this biological wealth. Our capacity to do so has increased enormously in recent times owing to new tools ranging from molecular approaches to remote-sensing capabilities of the biosphere from space. The numbers of scientists engaged in this broad research front are increasing, resulting in an explosion of new knowledge. In recent decades, with concerns about the mounting evidence of losses of biological diversity through human activities, a number of things have happened. One, it is becoming clear to everyone that our collective natural capital is being depleted. This concern has impinged on political processes at virtually all levels from local, to nation states, to international treaties. Many of these political processes have been fraught with difficulties, due in part to the complex nature of the problem—both scientifically but also because of difficulties at the science–policy interface.

Biodiversity scientists, particularly those who study natural systems in the field, find themselves in a very difficult position. In a sense, forces outside their control are randomly moving around the ‘equipment’ of their laboratories and in some instances even destroying the objects of their study. Of course, major changes to natural systems have always occurred because of natural cycles and disturbances. However, the rate of perturbation is now outside of the envelope of past experience. This new turmoil, or crisis, has focused attention on accelerating the job of the search for new knowledge but also in trying to understand and perhaps control the disruptive influences that are diverting their attention and productivity. There are some small upsides to this situation in that some of these disruptions are providing new insights to the forces that organize ecological systems as well as to the drivers of evolutionary response to change, such as those seen in the ecosystem responses to the introduction of new species into an area.

Of course, there are other concerns and enormous challenges stemming from these mounting losses, aside from the personal ones, where science can provide answers. A major question of the past decade has been, ‘what are the consequences of these losses in terms of the way of life that we lead’—ranging all the way from ethical and aesthetic concerns to the quality of water we drink and the food that we eat.

In the following, I consider how both the biodiversity science community and the relevant policy community are responding to this crisis. Are we moving fast enough in either sector to stem the tide of rapid deterioration of the Earth's natural capital? Can we do better?

I start with the policy response because it demonstrates, in part, the impediments we face because of inadequate policy formulation to deal with the changes that have incurred since international treaties were crafted nearly two decades ago.

## 2. The recent history of societal response to emerging environmental crises—things move very, very slowly

There has been concern for a long time regarding the loss of biological diversity through the activities of humans. A major turning point in public perception and subsequent policy action came through the publication of Rachel Carson's *Silent spring* (Carson 1962) that focused on the chemical pollution of landscapes by pesticides and their impacts on biota. This publication represented the beginnings of a global environmental movement and in the USA, led to such crucial legislation as the Endangered Species Act. Similarly, rapid deforestation in the species-rich tropical forests led to a new concern for the loss of biological diversity and the publication of a number of calls for action such as that of Eisner *et al*. (1981) pointing to ‘the silent crisis of our time: species extinction’ and the edited volume, *Biodiversity* (the first usage of this term), where it was noted that, ‘The current reduction of diversity seems destined to approach that of the great natural catastrophes at the end of the Paleozoic and Mesozoic eras—in other words, the most extreme in the past 65 million years’ (Wilson 1988). The Millennium Ecosystem Assessment (MA) (MA 2005) concluded that the most rapid loss in the history of the Earth's natural capital or ‘ecosystem services’ has occurred over the past 50 years, driven by the doubling of population and the increasing *per capita* consumption.

So, one can certainly make the case that science concerns sounded the alarm regarding the loss of biological diversity. These concerns led in turn to a formal international societal response to the biodiversity crisis at the UN Rio Convention in 1992 where the Convention on Biological Diversity (CBD) was crafted. At the same time, another looming crisis, global warming, led to the approval of a second international agreement, the Climate Convention. It is instructive to compare the success of these two conventions in achieving their stated aims as well as the process that led to their formulations. In the case of the Climate Convention, it was based on a science assessment performed by the Intergovernmental Panel on Climate Change (IPCC) that preceded the convention process (Bolin 2007). This assessment led to relatively precise goals for the convention, ‘The ultimate objective of this Convention is the stabilization of greenhouse gas concentrations in the atmosphere at a level that would prevent dangerous anthropogenic interference with the climate system’ (Article 2). In spite of this relatively precise language and three subsequent science assessments that have dramatically reduced the uncertainty that humans are driving these rapid changes, and in spite of the fact that CO_2_ increase in the atmosphere is still accelerating, we still have not agreed upon an international action to meet the objectives of this convention, although there are the beginnings of dramatic changes in individual practices in many parts of the world; however, these are driven, initially at least, more by the rising price of energy rather than the threat of global warming.

The CBD was not preceded by a science assessment and the language of the articles of the convention is not very precise although the objectives are grand, ‘ … the conservation of biological diversity, the sustainable use of its components and the fair and equitable sharing of the benefits arising out of the utilization of genetic resources’ (Article 2 on objectives). The teeth of the convention are framed in weak terms such as ‘Each Contracting Party shall, in accordance with its particular conditions and capabilities’ carry out the various articles, such as, ‘Develop national strategies, plans or programmes for the conservation and sustainable use of biological diversity … ’ (Article 6).

The CBD has its overriding principle that nations have ‘the sovereign right to exploit their own resources pursuant to their own environmental policies … .’ (Article 3). This principle has been the driving force for discussions within the Conference of Parties, and the second part of the principle hardly at all, that nations also have ‘ … the responsibility to ensure that activities within their jurisdiction or control do not cause damage to the environment of other States or of areas beyond the limits of national jurisdiction’. This latter part of the principle has not received much discussion but rather the main focus of attention has been on issues such as benefit sharing.

The focus on national sovereignty and jurisdiction by the CBD has left many important global biodiversity issues outside of the convention and has led to subsequent stop-gap measures such as the formation of a separate treaty to deal with the international issues relating to plant genetic resources for food and agriculture. This treaty was approved in 2001 and went into effect in 2004 (http://www.planttreaty.org/). However, other crucial global issues are not receiving adequate international policy attention, such as invasive species, and the biological diversity of the vast ocean areas beyond national jurisdiction. The open ocean is now treated as a commons and is suffering the consequences—with trawlers operating deeper and deeper.

Initial attempts to bring a science assessment into the convention process retroactively by the United Nations Environmental Programme (UNEP), the ‘Global Biodiversity Assessment’ (GBA) (UNEP 1995), was considered a scientific success, but was not particularly influential in the convention process because it took a global view of the problem rather than a country view where the convention was focused. There was a structural issue with the GBA in that it was not authorized by the parties of the convention and thus had no status for discussions within the Convention framework.

At the UN Rio +10 Conference in Johannesburg in 2002, the Millennium Development Goals were adopted, including Goal 7, to ensure environmental sustainability. One of the targets that was approved for this goal was to achieve a significant reduction in the rate of biodiversity loss as indicated by, among other things, the proportion of land covered by forests, the proportion of fish stocks within safe biological limits, the proportion of terrestrial and marine areas protected and the proportion of species threatened with extinction.

So, where are we in relation to the goals of the climate convention and the biological diversity convention in 2008? In spite of an enormous effort on part of the political and scientific communities and the important goals that have been set, there is no question that progress has been dismal. In terms of the climate convention, starting in 2000, the CO_2_ emissions into the atmosphere reached levels that were in excess of the worst-case scenarios produced by the IPCC (Raupach *et al*. 2007). The same is true for the melting of arctic ice (Stroeve *et al*. 2007).

We are moving into a very dangerous territory regarding more severe changes in the climate system. In relation to the CBD and the Millennium Development Goals, it is clear, with the exception of the target on an increase in protected areas, we will not come close to achieving the other agreed-upon targets, much less critical biodiversity indicators such as changes in population sizes and ranges. We continue to lose biodiversity at an alarming rate for those metrics for which we have data (e.g. fish stocks, where only data for commercial fisheries are available). We lack a system for quantifying the status of most dimensions of biodiversity, so it is difficult to see how much on track we are for achieving a significant reduction in biological diversity, but from data we do have the trends do not look promising.

So what are biodiversity scientists doing in this critical period in planetary history? Is the science moving fast enough to predict the outcomes on the complex interactions of biotic systems of rapid global changes that are occurring and that are already beyond the magnitudes of historical times?

The research community is continuing to make the case, with ever increasingly alarming numbers, that we are indeed losing our biotic resources. They are accelerating efforts to discover, catalogue and make widely available, information about the biotic richness on Earth, for example, the Global Biodiversity Information Facility. However, with the increasing losses that are being incurred, at all levels of biodiversity, scientists are also working on strategies to save or restore what we have but in the rapidly evolving context of global change. Then, they are working to find new ways of demonstrating the consequences of the massive losses of global natural capital to the welfare of society. I discuss each of these selected areas to give a sense of the inter-relationships of the road map that biodiversity community appears to be following.

## 3. The evidence for major losses—convincing and staggering

The following examples give some indications of the massive losses of biological diversity that are being incurred at all levels of organization, from genes, to species, to ecosystems and to entire landscapes. These examples illustrate the operational background for biodiversity scientists and the motivation for seeking new knowledge and approaches for stemming the wholesale degradation of the Earth's natural capital.

### (a) Genetics

In a global survey, Rischkowsky & Pilling (2007) reported that of 7616 livestock breeds included in their global survey, approximately 20 per cent were at risk and 62 breeds had gone extinct during the previous six years. For crops, Shand (1997) estimated that approximately 75 per cent of the genetic diversity of crops has been lost since the turn of the last century. In natural systems, Hughes & Daily (1997) estimate that millions of populations of wild species go extinct each year owing to land-use conversion. We now have powerful tools to assess genetic diversity, and no doubt as attention focuses on this issue we should have a more complete and precise picture of genetic degradation.

### (b) Species

The evidence for the loss of biodiversity at all levels is overwhelming and has been chronicled extensively (e.g. MA 2005; UNEP 2007; IUCN 2008). However, as discussed below, we seriously lack the capacity not only for enhanced discovery of the vast unknowns of diversity but also for how to chronicle the continuing status of those things we do know about and whose status needs monitoring for signs of degradation or recovery. Even though there are losses in diversity at virtually all levels of organization, as noted above, most of our attention has been directed towards the ‘end of the line’, that is, species extinctions. The data at this level are abundant, yet still inadequate to tell the full story. Those numbers we do have are, in many cases, sobering as indicated by the following recent examples:

### (c) Mammals

The distributional ranges of mammals, which have been identified as declining globally (173 species), have had their ranges reduced by 50 per cent, on average, since historic records of ranges have been documented (Ceballos & Ehrlich 2002). At the species level, according to the most recent IUCN Red List, nearly half of the world's primate species and subspecies are in danger of extinction (IUCN 2008).

### (d) Birds

Populations of many bird species are declining, In Europe, 45 per cent of once-common bird species are in decline. In North America, 20 once-common species have lost half of their populations (Birdlife International 2008).

### (e) Trees

According to calculations of Hubbell *et al*. (2008), a third of the Amazonian forest tree species will go extinct under projected land-use conversion during the next couple of decades. This calculation does not take into account possible climate change impacts.

### (f) Communities

#### (i) Ocean's oligotrophic waters

Areas of low chlorophyll concentrations in the surface waters have expanded by 15 per cent between 1998 and 2006, representing an area of some 6.6 km^2^, most probably related to the increased surface sea temperatures observed over this period (Polovina *et al*. 2008).

#### (ii) Coral reefs

Of 704 species of zooanthellate reef-building coral species that could be classified as to their conservation status (of the 845 total species), a third have an elevated risk of extinction driven by coral bleaching, diseases driven by sea-surface temperature increases and local disturbances. This is a dramatic increase in the extinction risk over the past 10 years (Carpenter *et al*. 2008). It is estimated that 50 per cent of the existing coral reefs are ailing and on the verge of collapse (Stone 2007).

### (g) Ecosystems and landscapes

Vast natural areas of the globe have been pre-empted to support the livelihoods of the growing human population. Over a third of the ice-free surface is devoted to livestock production and another 8 per cent to crop production used directly for human consumption (Steinfeld *et al*. 2006). The Earth's surface has been ‘sliced and diced’ through the vast network of transportation networks to support human activities, and the ‘plumbing system’, the rivers and streams, have been, in many areas of the world, utterly transformed by dams and diversions, all at great damage to many biotic systems.

These modifications have not been uniform over the globe, and certain regional ecosystems have been particularly impacted, such as Mediterranean forests and woodlands, temperate and tropical forests, woodlands and grasslands (MA 2005).

## 4. Game over

Given the lack of substantive progress on the climate and biodiversity conventions, as noted above, and the continued adverse trends that led to their formation, there is no wonder that some scientists are viewing the necessity to think beyond conservation as we normally think of it. This view is also driven by the lack of attention, and resulting consequences, to other substantive environmental issues such as overfishing, due in part to the lack of a treaty for the open oceans, in addition to the enormous changes in land use, which have large consequences not only for biodiversity but also for the climate system.

Many of the changes wrought by these drivers are already impacting ecosystems and in some cases, irreversibly so. These trends have resulted in the shift in thinking from the concentration on ‘mitigation’ of adverse drivers of change in order to protect the systems with which society has co-evolved to considering ‘adapting’ to the new ecosystems that are evolving under the new-world conditions. We are seeing this in the political world in addition to the scientific realm as indicated by the recent statement of Al Gore, ‘I used to think adaptation subtracted from our efforts on prevention. But I have changed my mind.’ (Gore 2008).

Some ecologists also think this. In many places in the world, we are beginning to see what are termed ‘novel or emerging ecosystems’ in response to new drivers. These are ecosystems where species occur in combinations and relative abundances that have not previously occurred. As noted above, one of the drivers of these changes is land degradation through human practices accompanied by the success of invasive species into these altered habitats (Hobbs *et al*. 2006). Hobbs *et al*. state that these systems are here to stay, and those efforts to restore them to ‘natural conditions’ are doomed to failure. Efforts, they note, are better given to managing these new systems to optimize the delivery of ecosystem services rather than to restoration.

Further, there are discussions on approaches for assisting species in adapting to the rapid changes that are already entrained. Hoegh-Guldberg *et al*. (2008) make the case that the rate of climate change will be so rapid during this next century that many species will not be able to adjust their distributions to new areas that would be suitable for their survival, noting that, ‘the future for many species and ecosystems is so bleak that assisted colonization might be their best chance’. They contend that we can do this safely if an adequate risk assessment and management plan is developed for each transplant candidate. Others maintain that the risk of creating invasive characteristics in the new environment, although small, is an issue (Mueller & Hellmann 2008) and that the details of carrying out such endeavours are fraught with policy roadblocks in addition to ecological concerns (Davidson & Simkanin 2008; Huang 2008).

The conservation community, more generally, has also taken a rather dramatic change in research direction in relatively recent times. It has only been in the past few years, as data have accumulated documenting the changes that have already occurred in the distribution of organisms, that the issue of climate change has come more to the fore in relation to reserve and corridor design, for example. A new sense of urgency is penetrating this community and discussions of the utilization of ‘triage’ approaches in the conservation of threatened species with differing points of view on the benefits of what some view as a last ditch approach (Bottrill *et al*. 2008) are taking place.

## 5. Can we do better?

There are those who counter the ‘game over’ argument by saying that we need ‘to marshal the financial, political and technical resources to stabilize the climate’ and to engage in effective management to build resilience into ecosystems (Schuttenberg & Hoegh-Guldberg 2007). The latter action is itself not the ‘business as usual’ conservation approach of reserves, however, because it involves active management. So, the logic now seems to be that even if we continue to work towards mitigation, as we must, we also have to be considering adaptive strategies to varying degrees to deal with future global changes.

We need also to work towards a better understanding by the scientific community, decision makers and the general public on the consequences of the losses of biodiversity that we have already incurred as well as that which is predicted for the near future but can nonetheless be avoided. One approach to building such knowledge is to view the many dimensions of biological diversity into a single integrated package. In the following, I indicate how the science community has developed this integrated view of biological diversity and the drivers of change that are degrading it. A large part of the problem that we face is that society is not sufficiently aware of the significance of the biodiversity losses that are occurring in terms of their own welfare. The emerging development of an ‘ecosystem-service’ approach seems to be gaining considerable momentum and has promise of making the linkage that is needed between biodiversity loss and degradation of human well-being that could revolutionize our collective view of the central role of nature in our lives.

## 6. An ecosystem-service approach

A couple of decades ago, as the view of the Earth as an integrated biophysical system was being formulated (Earth system science), the question asked was, what difference does biological diversity matter in Earth system dynamics? There was not a lot of evidence to bring to bear to answer that question because the research community had not focused on this rather fundamental question. Of course, there was a lot of knowledge about the role of organisms in relation to ecosystem productivity, hydrology and biogeochemistry, but not about the question of how important diversity, *per se*, was at any given taxonomic, or functional type, to ecosystem processes. Once this need was expressed, the research community responded rapidly and produced a wealth of information on the interplay between biological diversity and ecosystem functioning (Schulze & Mooney 1993; Kinzig *et al*. 2001; Tilman *et al*. 2001; Loreau *et al*. 2002; Gamfeldt & Hillebrand 2008). As is the case in most new research areas, the initial experiments to probe these relationships were simple in nature but with time they have increased in sophistication as the complexity of the issue has been revealed and the need for difficult experiments extended over a considerable time has been realized (Scherer-Lorenzen *et al*. 2005; Balvanera *et al*. 2006; Potvin & Gotelli 2008).

Similarly, in the most recent decade, another rather fundamental area was found to be lacking in knowledge, although there was an expressed need for this information from many sources. This was the relationship between the functioning of ecosystems and how these processes relate to human welfare. A very large international science assessment, the Millennium Ecosystem Assessment (MA 2005), was based, in part, on knowledge from this linkage, and yet the database needed to execute this assessment was rather depauperate. However, again, the science community responded to this lack of data with an enormous burst of creative activity, providing in a short amount of time a wealth of new knowledge that led to more than just connecting the dots in examining the crucial linkages between ecosystem functioning and services (Daily 1997). These extend from cataloguing the various services provided by species within a class of organisms, such as birds (Sekercioglu 2006), to quantifying the economic value of insects in providing pollination services (Kremen *et al*. 2007), to the role of the functioning of agricultural systems in providing services that are not widely appreciated (Swinton *et al*. 2007). In relation to the latter, the most recent FAO report on the state of food and agriculture focuses on paying farmers for services (FAO 2007).

There are increasing numbers of studies examining various ecosystem processes teasing apart what components of ecosystem functioning are delivering services (Brauman *et al*. 2007), how these can be mapped on landscapes (Naidoo *et al*. 2008) and how these services can be quantified as ‘production functions’ that can be used by economists (Polasky 2008). Also, studies have provided analytical techniques for determining what particular traits of species and communities, and their distribution, are the most important in providing services (Diaz *et al*. 2007). Probing these relationships is not limited to terrestrial ecosystems but also extends to marine systems (Worm *et al*. 2006).

Although there are plenty of examples of the science community responding quickly to a perceived lack of fundamental knowledge in an area of interest to their field and to society, progress is still slow in meeting the crisis of lack of knowledge noted above during a period of enormous recent past and rapidly accelerating depletion of the Earth's natural capital.

How do we speed things up and how do we set priorities? We are seeing a revolution in many institutions that recognize that probing deeply within any given knowledge sector is essential, but not alone sufficient, for making major science advances in many interface and integrated research areas that are crucial for societal welfare. We see this in many universities that are fostering interdisciplinary research as well as in research funding agencies that are creating bridging disciplinary area-funding opportunities, with some programmes insisting upon this research approach.

A particularly good example of this trend is in biological diversity research. Biological diversity is a complex area of knowledge and research as it focuses on diversity at many levels of knowledge and organization—from genes to landscapes, and including diversity of human cultures and practices. An integrated view of this research area was fostered by the Millennium Ecosystem Assessment, and thus it included both natural and social scientists working across a wide spectrum of knowledge that was crucial for not only understanding the nature and status of biodiversity, but also what is driving it in evolutionary time as well as in the recent history of human domination of the Earth.

**Figure 1.**
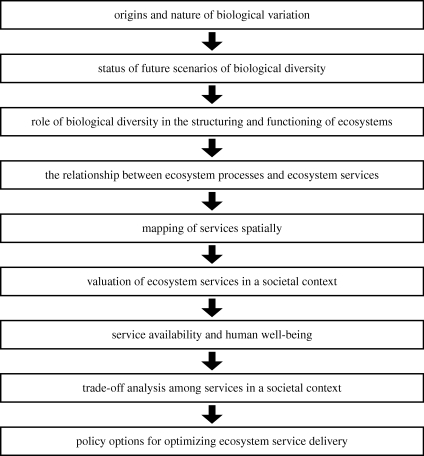
The chain of knowledge extending from basic science to policy application in the ecosystem-service paradigm. Teams of natural and social scientists are needed to provide integrated knowledge along this chain for any given area.

Among the blocks of knowledge that constitute the chain from basic understanding of the nature of diversity to the delivery of policy options regarding providing sustainable ecosystem services to society are those given in figure 1.

The links in the chain are all large and complex areas, many of them new to the research community. What characterizes this chain is that certain linkages need teams of natural and social scientists working together to make progress. One can enter at any level of this chain and of course make substantial contributions to fundamental knowledge and potentially to application. However, there are a number of reasons for pursing integrated research along the full chain. First, this brings together diverse partners from the natural and social sciences in order to unravel the complexities of drivers of change and biotic responses. Secondly, it reveals the crucial linkages, and dependencies, among the focal areas. Finally, it provides an integrated package of knowledge that can be easily translated to society, and hence to decision makers, on the crucial relevance of the components of biological diversity to everyday life. If these studies are locally based, as they must be in part, they can involve stakeholders and a wide range of knowledge providers and decision makers in the process.

The case can be made that the millions of species of organisms, and the genetic variation they contain, represent the self-replicating building blocks for the construction of ecosystems, which in turn can be viewed as factories that produce products through cycling carbon, water and nutrients in a renewable manner. These processes, along with the structural characteristics of ecosystems and the complex and diverse species interactions that occur within them, are the basis of ecosystem services, or the products or benefits that society derives from ecosystems. The Millennium Ecosystem Assessment (MA 2005) characterized these benefits into three major categories: provisioning (food, fresh water, fibre, etc.), regulating (flood control, disease control, water purification, etc.) and cultural services (aesthetic, spiritual, recreational, etc.). This brief statement encompasses a vast amount of new research that explores the linkages between diversity, on the one hand, and ecosystem functioning, on the other, extending from the experimental to the conceptual, as noted above.

Above, there was a brief discussion of the rapid progress being made among the first links in the chain from origins and nature of biodiversity to the valuation of services as indicated in figure 1. Regarding the valuation of services, this complex area is of course scale sensitive and is dependent on local value systems where non-economic values can assume high significance.

The linkage between ecosystem services and human well-being is critically important as was revealed in the MA where it was shown that maintaining ecosystem-service provisioning was crucial to meeting the UN Millennium Development Goals. In addition to the MA discussion (MA 2005), further progress in this area is given in Carpenter *et al*. (2009) and an International Council for Science, UNESCO, UN University report (ICSU UNESCO UNU 2008).

One of the most difficult areas in the ecosystem-service chain relates to trade-off analyses among services. Often, optimizing delivery of a given service may mean reduction of another, as in the well-illustrated example of enhancing the provisioning of food that can result in loss of clean water and the biotic systems that maintain this service. The problem with making adjustments to optimize trade-offs is that generally the institutions that should be involved in the trade-off discussions are separate entities and are competitive rather than cooperative, such as ministries of agriculture versus the environment, as a single example. This is absolutely a central impediment to rational decision making in the ecosystem-service arena and relates to the final link in the change designing optimal policy options for ecosystem delivery.

## 7. A couple of other pieces of the puzzle

**Figure 2.**
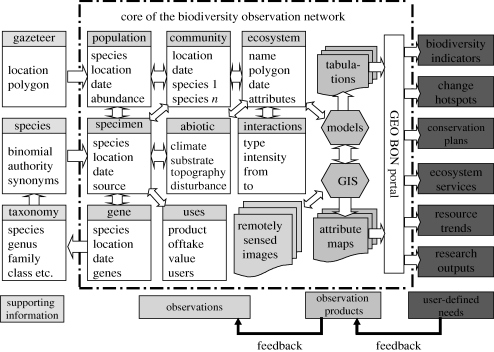
The structure of a proposed Global Biodiversity Observation System (Scholes *et al*. 2008).

As noted above, we do have statistics on the losses of biological diversity that have been incurred in recent times. The data come from many sources, often one-off analyses. We need to do much better not only in keeping track of what we have but also what we are losing in spatially explicit and quantitative terms. The lack of such data impedes our analysis of the losses of services that we are incurring and also impedes us from having a solid base for indicating how well we are doing in meeting targets that may be set. There is an important emerging effort to supply just such a system of book keeping of the status of our natural capital, this is the proposed Global Observation System of Systems in the task area of biodiversity (GEO-BON) as recently described by Scholes *et al*. (2008) (figure 2). What is important about this system is that it would seek input from the many diverse efforts to keep track of certain dimensions of biodiversity, such as species diversity or land cover, and from this provide output to the global community on integrated metrics such as indicators, hot spots, ecosystem services, etc.

The other piece is the establishment of an ongoing global and subglobal assessment process for biodiversity and ecosystem services. Single assessments such as the MA are valuable but not adequate to determine trends and to recognize new research areas that need attention in order to reduce uncertainty about our predictions regarding the status and fate of biological diversity and hence human welfare. Such an effort is now under discussion at the intergovernmental level and has been termed the Intergovernmental Science-Policy Platform on Biodiversity and Ecosystem Services (IPBES) and has been called for by the global scientific community (Diversitas and the Millennium Ecosystem Assessment community assisted by the UNEP).

## 8. Circling back to policy

At the beginning I noted that both the policy and scientific processes are moving too slowly to keep up with the rapid changes that are unfolding, leading to a view of despair by those who keep close track of these trends and unfavourable scenarios. It is quite evident that we will need a larger cadre of futurists among us who will be engaged in these issues who will be pondering aggressive solutions to the arising problems. This is already happening to a degree in the more frequent appearance of global geo-engineering schemes to avert global warming, which in turn evoke even greater angst regarding potential unintended consequences of such schemes. Nonetheless, we do need to have more scenario-building exercises for guidance, perhaps at a more local level where adaptive management can be used. The scenario-building area in biodiversity research is not well developed and needs more attention. As was found in the MA there is a lack of global models of biodiversity and hence ecosystem-service responses to various drivers of change.

At the same time as we are on the edge looking forward, we need to use a better framework that can give the policy community some clear guidelines and a rationale for protecting biodiversity. The ecosystem-service paradigm is one such rationale that could easily be incorporated into policy instruments with clear goals of protecting given ecosystem services at a specific level for human welfare. In any event, it is time to revisit the CBD to make it more effective and to give it some clear guidelines for action. As for speeding up the scientific process—it is remarkable how quickly the science community has built a whole new structure as noted above; however, there are many things that are yet to be tackled and need urgent attention. Funds by agencies and foundations directed towards a given research area generally result in a spike in attention. However, an important driver for attention is an understanding within the larger community of the urgent need for knowledge, and policy, to help society over the crisis that we all face.
